# Supplementary material for: Insights on the impact of mitochondrial organisation on bioenergetics in high-resolution computational models of cardiac cell architecture
Source: PLoS Comput Biol. 2018 Dec 5;14(12):e1006640. doi: 10.1371/journal.pcbi.1006640 (PMC6296675; doi:10.1371/journal.pcbi.1006640)
Supplement: S1 Fig — (A) Model predicted distribution of oxygen in cross section 1 presented in grayscale image. (B) Resolution of the image predicted by the model was reduced by a margin of 5 times. (C) Subsequently, we applied a point spread function over the image, followed by application of poison noise, to derive the simulated confocal microscope image at low resolution. (PDF) [file pcbi.1006640.s001.pdf]

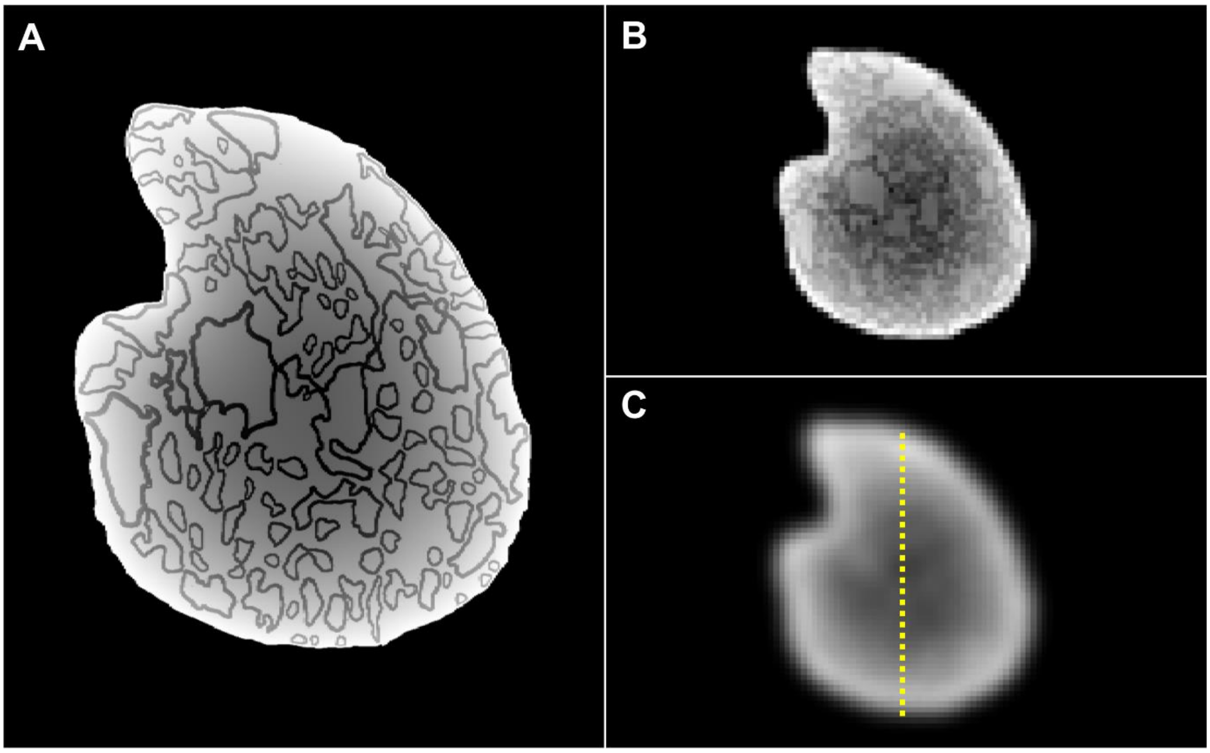

S1 Fig. Simulation of confocal microscope images from model predicted species distribution. (A) Model predicted distribution of oxygen in cross section 1 presented in grayscale image. (B) Resolution of the image predicted by the model was reduced by a margin of 5 times. (C) Subsequently we applied a point spread function over the image, followed by application of poison noise, to derive the simulated confocal microscope image at low resolution.
