# Supplementary material for: Insights on the impact of mitochondrial organisation on bioenergetics in high-resolution computational models of cardiac cell architecture
Source: PLoS Comput Biol. 2018 Dec 5;14(12):e1006640. doi: 10.1371/journal.pcbi.1006640 (PMC6296675; doi:10.1371/journal.pcbi.1006640)
Supplement: S2 Fig — The black and green lines correspond to the total area fraction of mitochondria and nucleus present in each cross section of the cell. The first and third quartile of the mitochondrial density distribution in each cross section is represented using the lower and upper bound of the light blue area. There is negligible difference between the mitochondrial area fraction and quartile values of mitochondrial density distribution corresponding to different cross sections. (PDF) [file pcbi.1006640.s002.pdf]

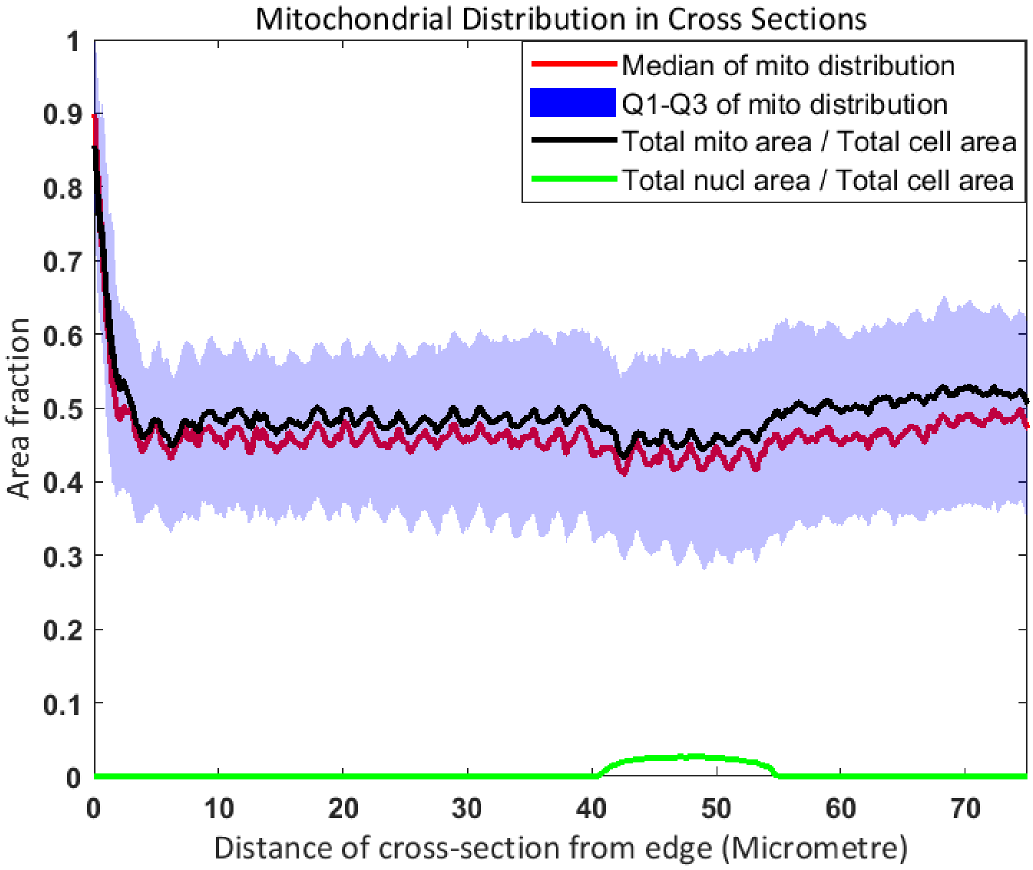

S2 Fig. Spatial variation in distribution of mitochondria and nuclei along the longitudinal axis of the cell. The black and green lines correspond to the total area fraction of mitochondria and nucleus present in each cross section of the cell. The first and third quartile of the mitochondrial density distribution in each cross section is represented using the lower and upper bound of the light blue area. There is negligible difference between the mitochondrial area fraction and quartile values of mitochondrial density distribution corresponding to different cross sections.
