# Supplementary material for: Insights on the impact of mitochondrial organisation on bioenergetics in high-resolution computational models of cardiac cell architecture
Source: PLoS Comput Biol. 2018 Dec 5;14(12):e1006640. doi: 10.1371/journal.pcbi.1006640 (PMC6296675; doi:10.1371/journal.pcbi.1006640)
Supplement: S1 Text — (PDF) [file pcbi.1006640.s003.pdf]

S1 Text

This supporting information presents a detailed description of the partial differential equations (PDE) based finite element model used to simulate the results in the main paper.

1. Introduction

The supporting information presents a detailed description of the partial differential equations (PDE) based finite element model used to simulate the results in the main paper. The model consists of total 17 state variables as described in table 1.

Section 2 introduces a set of 7 PDEs and 12 algebraic equations (AE) used for modelling the myofibrilar reactions and force dynamics Following this, section 3 presents a set of 7 PDEs and 12 AEs used for modelling the transport fluxes through the mitochondrial outer membrane (MOM). It further contains a detailed description of 10 ODEs and 12 AEs used to simulate the state variables corresponding to mitochondrial electron transfer chain (ETC) and other transport fluxes through the mitochondrial inner membrane (MIM). Finally, Section 4 presents the simulation protocol utilized to calculate the force dynamics corresponding to the metabolite distribution in the myofibrils.

Table 1: State variables of the system

State Variable	Definition (Unit)	Location
ATP	ATP concentration (µM)	Entire cell
MgATP	Mg bound ATP concentration (µM)	Entire cell
ADP	ADP concentration (µM)	Entire cell
MgADP	Mg bound ADP concentration (µM)	Entire cell
AMP	AMP concentration (µM)	Myofibril and IMS regions
PCr	Phosphocreatine concertation (µM)	Myofibril and IMS regions
Cr	Creatine concertation (µM)	Myofibril and IMS regions
Pi	Inorganic phosphate concertation (µM)	Entire cell
O_2	Oxygen concentration (µM)	Entire cell
H^+	H^+ concentration (also expressed in pH)	Entire cell
K^+	Potassium ion concentration (µM)	Mitochondrial matrix
Mg^{2+}	Free magnesium ion concentration (µM)	Entire cell
NADH	NADH concertation (µM)	Mitochondrial matrix
NAD	NAD concertation (µM)	Mitochondrial matrix
Q	Ubiquinone concertation (µM)	Mitochondrial matrix
QH_2	Ubiquinol concertation (µM)	Mitochondrial matrix
Cred	Cytochrome C (reduced) concertation (µM)	IMS regions
Cox	Cytochrome C (oxidized) concertation (µM)	IMS regions
$\Delta\psi$	Mitochondrial membrane potential (mV)	Mitochondrial inner membrane
F_{peak}	Peak twitch force produced corresponding to a single transient signal of calcium (Ca^{2+})	Myofibril
t_{twitch}	Duration of twitch when the force is above 5% of Fpeak (ms)	Myofibril

2. Simulating the reactions at myofibrils–

Following differential equations represent the state variables in the myofibrilar region of the cell. The state variables shaded in light grey are either calculated using PDEs or using linear algebraic equations. However, the state variables shaded in slightly higher tone are assumed to have a constant value throughout the cell cross-sections.

$$\frac{dATP}{dt} = D_{ANP}\nabla^2 ATP - v_{CK} + v_{AK} - v_{ATPase} \qquad \frac{dADP}{dt} = D_{ANP}\nabla^2 ADP + v_{CK} - 2v_{AK} + v_{ATPase}$$

$$\frac{dPi}{dt} = D_{Pi}\nabla^2 Pi + v_{ATPase} \qquad \frac{dAMP}{dt} = D_{ANP}\nabla^2 AMP + v_{AK}$$

$$\frac{dPCr}{dt} = D_{PCr}\nabla^2 PCr + v_{CK} \qquad \frac{dCr}{dt} = D_{Cr}\nabla^2 PCr - v_{CK}$$

$$\frac{dO_2}{dt} = D_{O_2}\nabla^2 O_2 \qquad MgATP = \frac{ATP.Mg^{2+}}{KDT + Mg^{2+}} \qquad MgADP = \frac{ADP.Mg^{2+}}{KDD + Mg^{2+}}$$

$$pH = 7.1 \qquad K^+ = 0.15e+06\ \mu M \qquad Mg^{2+} = 1.0e+03\ \mu M$$

Here, v_{ATPase} denotes the rate of ATP consumption at any particular point of the myofibril, and it is modelled as a function of the concentration of the ATP, ADP and Pi present at this spatial point.

$$v_{ATPase} = \frac{X_{ATPase}}{1 + R\dfrac{ADP.Pi}{ATP}}$$

The detailed description of various reaction rates used in these equations can be found in Table 2. Table 3 presents the definition and values of model constants used in these equations.

Table 2: Fluxes of myofibrilar reactions

Symbol	Flux (M/sec)	Source of model implemented
v_{ATPase}	Flux of ATP consumption in myofibril	Wu et al. [1]
v_{CK}	Flux of creatine kinase reaction	Vendelin et al. [2]
v_{AK}	Flux of adenylate kinase reaction	

Table 3: Definition of model constants

Symbol	Species	Value	Source
D_{ANP}	Diffusivity of ATP, ADP and AMP	145 μm^2/s	Vendelin et al. [2]
D_{PCr}, D_{Cr}	Diffusivity of PCr and Cr	260 μm^2/s	
D_{Pi}	Diffusivity of inorganic phosphate	327 μm^2/s	
D_{O_2}	Diffusivity of oxygen	2410 μm^2/s	Beard et al. [3]
r_{ANP}	Reduction in diffusivity of ATP, ADP and AMP at IMS space	0.01	Aliev et al [4].
PCr_{total}	Total concentration of PCr and Cr in myofibrils and IMS	23 mM	Vendelin et al. [2]
ANP_{total}	Total concentration of ATP and ADP in the cell	10 mM	
KDT	Mg^{2+} dissociation constant for myofibrilar ATP	24.0 μM	
KDD	Mg^{2+} dissociation constant for myofibrilar ADP	347.0 μM	
$KDTm$	Mg^{2+} dissociation constant for mitochondrial ATP	17.0 μM	
$KDDm$	Mg^{2+} dissociation constant for mitochondrial ADP	282.0 μM	
NAD_{total}	Total matrix NAD(H) concentration	2970.0 μM	
Q_{total}	Total matrix ubiquinol concentration	1350.0 μM	
x_{buff}	Constant representing buffering capacity of the matrix space	100 M^{-1}	

C_{total}	Total IMS cytochrome C concentration	2700.0 µM	Beard [5]
C_{IMS}	Capacitance of inner membrane	1.0e-06 M/Litre of mitochondria/mV	
V_1	maximal MiCK reaction rates in forward direction	0.008 mol/s/Litre of Mitochondria	Aliev et al. [4]
V_{-1}	maximal MiCK reaction rates in reverse direction	0.00035 mol/s/Litre of Mitochondria	
W_M	water volume per total mitochondrial volume	0.72376	Beard [5]
W_{IMS}	IMS water volume per total mitochondrial volume	0.1	
W_X	Matrix water volume per total mitochondrial volume	0.9	

Boundary Conditions: As we used PDEs to describe the reactions in myofibril, it also necessitated the application of suitable boundary conditions corresponding to the PDEs. We assumed that there is no transportation of metabolites like ATP, ADP, AMP, Pi, PCr and Cr across the cell membrane of the 2D cross sections. This led to the application of neumann boundary conditions with zero flux across the cell membrane. For the PDE describing diffusion of O_2, we imposed a dirichilet boundary condition with a uniform O_2 concentration across the whole length of cell membrane.

3. Simulating the reactions at mitochondrial IMS and matrix regions –

Transportation of metabolites between the IMS and myofibril – Transportation of various metabolites between the IMS and myofibril is modelled using simple diffusion. Following partial differential equations represent the state variables in the IMS region of the cell. Detailed description of all the variables used in these equations can be found in table 4, while table 3 lists the model constants and their values.

$$\frac{dATP}{dt} = r_{ANP} * D_{ANP}\nabla^2 ATP + (-v_{MICK} + v_{MIAK} + v_{ANT})/W_{IMS}$$

$$\frac{dADP}{dt} = r_{ANP} * D_{ANP}\nabla^2 ADP + (v_{MICK} - 2\, v_{MIAK} - v_{ANT})/W_{IMS}$$

$$\frac{dAMP}{dt} = r_{ANP} * D_{ANP}\nabla^2 AMP + v_{MIAK}/W_{IMS} \qquad \frac{dPCr}{dt} = D_{PCr}\nabla^2 PCr + v_{MICK}/W_{IMS}$$

$$\frac{dPi}{dt} = D_{Pi}\,\nabla^2 Pi - v_{PiH}/W_{IMS} \qquad \frac{dCr}{dt} = D_{Cr}\nabla^2 PCr - v_{MICK}/W_{IMS}$$

$$\frac{dO_2}{dt} = D_{O_2}\nabla^2 O_2 \qquad MgATP = \frac{ATP.Mg^{2+}}{KDTm + Mg^{2+}} \qquad MgADP = \frac{ADP.Mg^{2+}}{KDDm + Mg^{2+}}$$

$$pH = 7.1 \qquad K^+ = 0.15e+06\ \mu M \qquad Mg^{2+} = 1.0e+03\ \mu M$$

Table 4: Fluxes of mitochondrial reactions

Symbol	Flux (mol/sec/Litre of mitochondria)	Source of model implemented
v_{DH}	Dehydrogenase flux representing the TCA cycle and other NADH-producing reactions	Beard [5]
v_{C1}, v_{C3}, v_{C4} and v_{C5}	Flux through complex I, complex III, complex IV and complex V (F_1F_0 - ATP synthase)	
v_{leak}	Flux of proton leak across the inner membrane	
v_{ANT}	Rate of exchange of metabolites through the adenine nucleotide translocases (ANT) channels	
v_{PiH}	Flux through Phosphate Hydrogen co-transporter	
v_{KH}	Flux through K^+/ H^+ antiporter	
v_{MICK}	Flux of mitochondrial creatine kinase reaction	

v_{MIAK}	Flux of mitochondrial adenylate kinase reaction	Vendelin et al. [2]

To represent the mitochondrial creatine kinase (mtCK) reaction in the model, we used a modified version of the mtCK reaction equation developed by Venedelin et al. In their study it was assumed that ANT channels present in the mitochondrial inner membrane releases the ATP from the matrix into a narrow micro compartment in the IMS. In the current study we did not consider the existence of micro-compartments separate from the rest of the IMS and accordingly modified the equation as –

$$v_{MICK} = \frac{V_1 \frac{MgATP * Cr}{(k_{ia} * k_b)} - V_{-1} \frac{MgADP * PCr}{(k_{ic} * k_d)}}{den_{MICK}}$$

Here, V_1 and V_{-1} denotes the maximal CK reaction rates in forward and reverse directions. The details of various dissociation constants used in this equation can be found in vendelin et al [2].

Reactions representing Oxidative phosphorylation –

Every node point in the IMS was considered to be metabolically coupled with matrix of individual mitochondria through the following ODE equations. These ODEs describe the action of various electron transfer chain complexes (Complex I-IV) and channels like adenine nucleotide translocator.

$$\frac{dNADH}{dt} = (v_{DH} - v_{C1})/W_X \qquad \frac{dQH_2}{dt} = (v_{C1} - v_{C3})/W_X$$

$$NAD = NAD_{total} - NADH \qquad Q = Q_{total} - QH_2 \qquad Cox = C_{total} - Cred$$

$$\frac{dCred}{dt} = 2(v_{C3} - v_{C4})/W_{IMS} \qquad \frac{dO_2}{dt} = -v_{C4}/(2 * W_{IMS})$$

$$\frac{dH^+}{dt} = x_{buff} * H^+ * (v_{DH} - 5v_{C1} - 2v_{C3} - 4v_{C4} + (n_A - 1) * v_{C5} + 2v_{PiH} + v_{leak} - v_{KH})/W_X$$

$$\frac{d\Delta\psi}{dt} = (4v_{C1} + 2v_{C3} + 4v_{C4} - n_A v_{C5} - v_{ANT} - v_{leak})/C_{IMS}$$

$\frac{dATP}{dt} = (v_{C5} - v_{ANT})/W_X$	$\frac{dADP}{dt} = (v_{ANT} - v_{C5})/W_X$	
$MgATP = \frac{ATP.Mg^{2+}}{KDTm + Mg^{2+}}$	$MgADP = \frac{ADP.Mg^{2+}}{KDDm + Mg^{2+}}$	$Mg^{2+} = 3.8e + 02\ \mu M$
$\frac{dPi}{dt} = (v_{PiH} - v_{C5})/W_X$	$\frac{dK^+}{dt} = v_{KH}/W_X$	

4. Simulation protocol utilized to calculate the force dynamics

The force dynamics across the myocyte cross-section was simulated using the metabolite-sensitive Tran et. al. [6] model of cross-bridge kinetics. At each node point, an isometric twitch was simulated based on the distribution of metabolite concentrations at the node. Each isometric twitch was activated by a known Ca2+ transient. The equation for the Ca2+ transient is detailed in Rice et. al [7] and the parameters used in this study were $\tau_1 = 20$ ms, $\tau_2 = 5.5$ ms, $Ca_{\text{amplitude}} = 1.2\ \mu$M, $Ca_{\text{diastolic}} = 0.09\ \mu$M.

For each isometric twitch simulated at each of the nodes, the peak twitch force (F_{peak}) and the duration of the twitch above 5% of peak twitch force (t_{95}) was calculated.

5. References

1. Wu F, Zhang EY, Zhang J, Bache RJ, Beard DA. Phosphate metabolite concentrations and ATP hydrolysis potential in normal and ischaemic hearts. The Journal of physiology. 2008;586(17):4193-208.
2. Vendelin M, Kongas O, Saks V. Regulation of mitochondrial respiration in heart cells analyzed by reaction-diffusion model of energy transfer. American Journal of Physiology - Cell Physiology. 2000;278(4):C747-C64.
3. Beard DA, Bassingthwaighte JB. Modeling Advection and Diffusion of Oxygen in Complex Vascular Networks. Ann Biomed Eng. 2001;29(4):298-310. doi: 10.1114/1.1359450.
4. Aliev MK, Saks VA. Compartmentalized energy transfer in cardiomyocytes: use of mathematical modeling for analysis of in vivo regulation of respiration. Biophysical Journal. 1997;73(1):428-45. doi: http://dx.doi.org/10.1016/S0006-3495(97)78082-2.
5. Beard DA. A Biophysical Model of the Mitochondrial Respiratory System and Oxidative Phosphorylation. PLoS Comput Biol. 2005;1(4):e36. doi: 10.1371/journal.pcbi.0010036.
6. Tran K, Smith NP, Loiselle DS, Crampin EJ. A metabolite-sensitive, thermodynamically constrained model of cardiac cross-bridge cycling: implications for force development during ischemia. Biophysical journal. 2010;98(2):267-76.
7. Rice JJ, Wang F, Bers DM, de Tombe PP. Approximate Model of Cooperative Activation and Crossbridge Cycling in Cardiac Muscle Using Ordinary Differential Equations. Biophysical Journal. 95(5):2368-90. doi: 10.1529/biophysj.107.119487.
